# Editorial: Glycosylation Changes in Cancer: An Innovative Frontier at the Interface of Cancer and Glycobiology

**DOI:** 10.3389/fonc.2016.00254

**Published:** 2016-11-28

**Authors:** Leonardo Freire-de-Lima, Jose Osvaldo Previato, Lucia Mendonça-Previato

**Affiliations:** ^1^Laboratório de Glicobiologia, Instituto de Biofísica Carlos Chagas Filho, Universidade Federal do Rio de Janeiro, Rio de Janeiro, Brazil

**Keywords:** cancer, metastasis, glycosylation, glycoconjugates, tumor glycobiomarkers, lectins

**The Editorial on the Research Topic**

**Glycosylation Changes in Cancer: An Innovative Frontier at the Interface of Cancer and Glycobiology**

Over the last 10 years, important discoveries have been made in the individual disciplines of glycoscience, which have contributed to the deciphering of numerous human diseases, particularly the different types of cancer. It is well documented that malignant transformation and cancer progression result in fundamental changes in the glycosylation patterns of cell surface glycoconjugates. Such changes are able to govern essential molecular phenomena in cell biology. Examples include (i) cell signaling pathways, (ii) cell–cell and/or cell-extracellular matrix (ECM) interactions, (iii) cell invasion, (iv) tumor angiogenesis, (v) immunomodulation, and (vi) metastasis formation. The research topic “Glycosylation Changes in Cancer: An Innovative Frontier at the Interface of Cancer and Glycobiology” provides invaluable information on the exciting field of oncoglycobiology, where one original research and outstanding reviews are presented.

In their original article, Mihalache et al. addressed the differential expression of mucins and the expression level of the major *O*-linked glycans in human colorectal cancer (CRC). The authors described differences in mucin glycosylation between CRC of various stages in order to propose new predictive or prognostic biomarkers.

Kolbl et al. discussed changes in the glycosylation pattern in breast cancer. The authors proposed that such aberrant glycan structures expressed by transformed breast cancer cells might be useful for diagnosis, determination of tumor stage, and prognosis but can as well be targets for therapeutic strategies.

In their opinion article, Nagarajan et al. highlighted the mechanisms by which heparin sulfate proteoglycans (HSPGs), such as glypicans, syndecans, and perlecans, are able to modulate tumor growth and metastasis.

Daniotti et al. provided a general overview on the metabolism of glycolipids, both in normal and tumor cells, as well as examining glycolipid-mediated immune modulation and the main successes achieved in immunotherapies using gangliosides as molecular targets.

Baldini and Lefebvre discussed about the reciprocal regulation of enzymes that drive lipogenesis (acetyl co-enzyme A carboxylase and fatty acid synthase) and *O*-GlcNAc transferase. In this perspective article, the authors pave the way for the exploration of the intricate functions of these actors that play a central role in tumor growth and spreading.

Li et al. summarized how glycans might influence cancer cells undergoing epithelial–mesenchymal transition (EMT), a biological process that allows a polarized epithelial cell, which normally interacts with basement membrane *via* its basal surface, to undergo multiple biochemical changes that enable it to assume a mesenchymal cell phenotype. Examples include (i) enhanced migratory capacity, (ii) invasiveness, (iii) elevated resistance to apoptosis, and (iv) greatly increased production of ECM components.

Pakkiriswami et al. outlined recent advances in our understanding of glycosylation of Notch proteins and the impact of altered Notch glycans in promoting tumorigenesis and metastasis. The authors defend the hypothesis that a full understanding of how atypical glycan structures affect Notch function requires further analysis. Methods need to be developed to examine how such changes in glycosylation temporally and spatially modulate cancer progression.

Mereiter et al. presented an overview on the main glycosylation changes in gastrointestinal cancers and the role of glycosylation as a modulator of the function of several key players in cancer cell biology. The authors also addressed several state-of-the-art techniques currently applied in this field, such as glycomic and glycoproteomic analyses, and provided an outlook to future perspectives and clinical applications.

Nardy et al. addressed how tumor-associated carbohydrate antigens (TACAs) confer to the cancer cells, the ability of dissemination by escaping the immunesurveillance, and thereby affect cancer progression.

Sindrewicz et al. summarized the current understanding concerning the molecular mechanisms underlying increased expression of the oncofetal Thomsen–Friedenreich disaccharide Galβ1-3GalNAc (TF antigen) in cancer, especially the structural nature and biological impact of TF interaction with galectins, in particular galectin-1 and galectin-3, on cancer progression and metastasis.

In their review article, Natoni et al. proposed that the inhibition of selectin–selectin ligand interactions might be useful to improve the treatment of cancer patients. However, the consequence of this strategy will depend on the type of cancer and the stage at diagnosis. Strategically, the authors suggest that the impairment of selectin/selectin ligand interactions may be achieved by the inhibition of key enzymes responsible for generating the selectin ligands, such as sialyltransferases (STs), or blockage of selectin/selectin ligands interactions by the use of a variety of compounds, such as glycomimetics, heparin derivatives, and blocking antibodies.

Taparra et al. reviewed the potential role of the hexosamine biosynthetic pathway (HBP) in driving EMT-related cancer processes. The authors believe that expanding the knowledge of how changes in metabolic pathways observed in cancer (e.g., the HBP) impact the distribution and composition of glycoconjugates may provide deeper insights into mechanisms of cancer biology.

Cagnoni et al. addressed the current status of the discovery and development of chemical lectin inhibitors and discussed novel strategies to limit cancer progression by targeting lectin–glycan interactions. In addition, the authors outlined the importance of understanding the biochemistry, biology, and glycobiology involved in tumor development and progression. They claim that the major challenge faced by this field is designing selective inhibitors of lectin–glycan interactions with increased bioavailability, what would improve cancer treatments.

Cardoso et al. highlighted the impact of galectin–glycan interactions in the tumor microenvironment, especially about the way that galectin-3 may participate in the activation of mitogen-activated protein kinases (MAPK) signal transduction pathways, leading to cancer cell proliferation, and resistance to apoptosis.

Finally, Fonseca et al. proposed that glycosylation changes in cancer cells might connect the multidrug resistance (MDR) phenotype and the EMT process, two multifactorial events able to modulate cancer cell half-life and tumor growth and metastasis.

Taken together, all articles published in this research topic reinforce the notion that oncoglycobiology is an important biological and medical field, and further research in this lively area may render valuable information, which might be used for therapeutic and diagnostic purposes.

## Author Contributions

LF-d-L, JP, and LM-P wrote the paper. All the authors read and approved the final version of the manuscript.

## Conflict of Interest Statement

The authors declare that the research was conducted in the absence of any commercial or financial relationships that could be construed as a potential conflict of interest.

